# Synthesis, Biological Evaluation, and Molecular Modeling of Aza-Crown Ethers

**DOI:** 10.3390/molecules26082225

**Published:** 2021-04-12

**Authors:** Stepan S. Basok, Igor A. Schepetkin, Andrei I. Khlebnikov, Anatoliy F. Lutsyuk, Tatiana I. Kirichenko, Liliya N. Kirpotina, Victor I. Pavlovsky, Klim A. Leonov, Darya A. Vishenkova, Mark T. Quinn

**Affiliations:** 1 A.V. Bogatsky Physico-Chemical Institute of National Academy of Science of Ukraine, 65080 Odessa, Ukraine; stepan_basok@ukr.net (S.S.B.); lutsyuk@ukr.net (A.F.L.); ti.kirichenko@ukr.net (T.I.K.); 2 Department of Microbiology and Cell Biology, Montana State University, Bozeman, MT 59717, USA; schepetkin@yahoo.com (I.A.S.); liliya.kirpotina@montana.edu (L.N.K.); 3 Kizhner Research Center, National Research Tomsk Polytechnic University, Tomsk 634050, Russia; aikhl@chem.org.ru (A.I.K.); victor_pavlovsky@mail.ru (V.I.P.); vishenkova_darya@mail.ru (D.A.V.); 4 Innovative Pharmacology Research, LLC, Tomsk 634021, Russia; leonov_k90@mail.ru

**Keywords:** aza-crown ether, neutrophil, ionophore, density-functional theory (DFT), quantitative structure-activity relationship (QSAR) modeling

## Abstract

Synthetic and natural ionophores have been developed to catalyze ion transport and have been shown to exhibit a variety of biological effects. We synthesized 24 aza- and diaza-crown ethers containing adamantyl, adamantylalkyl, aminomethylbenzoyl, and ε-aminocaproyl substituents and analyzed their biological effects in vitro. Ten of the compounds (**8**, **10**–**17**, and **21**) increased intracellular calcium ([Ca^2+^]_i_) in human neutrophils, with the most potent being compound **15** (*N*,*N*’-*bis*[2-(1-adamantyl)acetyl]-4,10-diaza-15-crown-5), suggesting that these compounds could alter normal neutrophil [Ca^2+^]_i_ flux. Indeed, a number of these compounds (i.e., **8**, **10**–**17**, and **21**) inhibited [Ca^2+^]_i_ flux in human neutrophils activated by *N*-formyl peptide (*f*MLF). Some of these compounds also inhibited chemotactic peptide-induced [Ca^2+^]_i_ flux in HL60 cells transfected with N-formyl peptide receptor 1 or 2 (FPR1 or FPR2). In addition, several of the active compounds inhibited neutrophil reactive oxygen species production induced by phorbol 12-myristate 13-acetate (PMA) and neutrophil chemotaxis toward *f*MLF, as both of these processes are highly dependent on regulated [Ca^2+^]_i_ flux. Quantum chemical calculations were performed on five structure-related diaza-crown ethers and their complexes with Ca^2+^, Na^+^, and K^+^ to obtain a set of molecular electronic properties and to correlate these properties with biological activity. According to density-functional theory (DFT) modeling, Ca^2+^ ions were more effectively bound by these compounds versus Na^+^ and K^+^. The DFT-optimized structures of the ligand-Ca^2+^ complexes and quantitative structure-activity relationship (QSAR) analysis showed that the carbonyl oxygen atoms of the *N*,*N*’-diacylated diaza-crown ethers participated in cation binding and could play an important role in Ca^2+^ transfer. Thus, our modeling experiments provide a molecular basis to explain at least part of the ionophore mechanism of biological action of aza-crown ethers.

## 1. Introduction

Crown ethers are macrocyclic polyethers containing three to twenty oxygen atoms separated by two or more carbon atoms, which can be either substituted or unsubstituted. Structurally, crown ethers possess a hydrophobic ring surrounding a hydrophilic cavity, which enables them to form stable complexes with metal ions, and at the same time to be incorporated in the lipid fraction of the cell membrane. Consequently, they exhibit ionophore properties by facilitating ion transport across membranes down their electrochemical gradients [1]. Thus, they behave similarly to natural ionophores, such as valinomycin, nigericin, and monensin.

Since the discovery of the crown ethers over 50 years ago [2], the chemistry of synthetic macrocycles for highly selective, structure-specific, supramolecular (host/guest) complexation has experienced rapid development for various applications, including the development of optically active sensors and mobile ion carriers [3,4,5,6]. Synthetic ionophores or carriers have also been developed as simplified models to study the driving factors for ion translocation. For example, a variety of tailored crown ethers have been successfully used as molecular transporters that span the lipid bilayer (e.g., [7]). These macrocycles are exceptionally versatile and can selectively bind to a range of metal ions and a variety of neutral and ionic organic species [8,9].

Crown ethers also have potential in the development of novel therapeutics. For example, crown ethers with antitumor activity include DNA intercalators and actinomycin D [10,11]. Aza- and diaza-crown ethers are of particular interest because their biological properties can be modified by introducing various substituents on their nitrogen atoms. For example, complexes of diaza-18-crown-6 with salts of arylchalcogen acetic acids, as well as glycyl derivatives of diaza-18-crown-6, have been shown to exhibit anti-proliferative activity [12,13]. Likewise, benzodiaza-15-crown-5 compounds containing two carboxymethyl or 3-(carboxy)prop-1-yl substituents on their nitrogen atoms were antimutagenic [14]. Addition of an adamantyl group to diaza-18-crown-6 resulted in optimal membrane permeability and conformational constraint for efficient transport through lipid membranes [15], facilitating its antitumor activity [16,17].

Crown ether derivatives have also been reported to exhibit other biological properties. For example, complexes of diaza-crown ethers with Fe_3_O_4_ have been shown to be active against Gram-positive and Gram-negative microorganisms [18]. Likewise, diaza-18-crown-6 lariate compounds have been shown to have antimicrobial activity and also significantly increase the effectiveness of rifampicin and tetracycline [19,20]. Benzyl derivatives of diaza-18-crown-6 ethers have been shown to exhibit cardiotropic effects [21], whereas naphthyl derivatives of diaza-crown ethers have been shown to have hematopoietic and colony-stimulating activity [22]. Moreover, some aza-15-crown-5 ether derivatives have been reported to have nootropic, antihypoxic, antiamnesic, and antiviral activities [23,24,25,26].

Little information regarding the immunomodulatory activity of crown ethers has been reported, aside from one publication indicating that dibenzo-18-crown-6 ethers have anti-inflammatory activity [27]. On the other hand, the natural ionophores valinomycin, nigericin, and monensin have been shown to modulate neutrophil functional activity [28,29,30,31,32,33]. Here, we elucidated the biological activity of structurally related adamantyl-containing aza- and diaza-crown compounds and found that they elevated the cytosolic Ca^2+^ concentrations ([Ca^2+^]_i_) in human neutrophils and also modulated *N*-formyl peptide receptor (FPR)-dependent functional activity. Quantum chemical calculations and molecular modeling were performed on five structurally-related diaza-crown ethers and their complexes with Ca^2+^, Na^+^, and K^+^ to obtain a set of molecular electronic properties and to correlate these properties with biological activity.

## 2. Results and Discussion

### 2.1. Synthesis of Crown Ethers

**Scheme 1:**
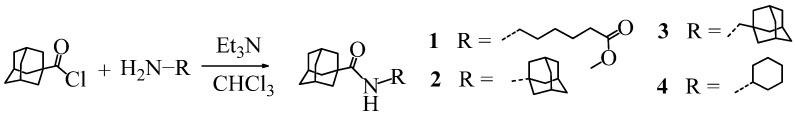
General method for the synthesis of adamantylamides **1**–**4**.

**Scheme 2:**
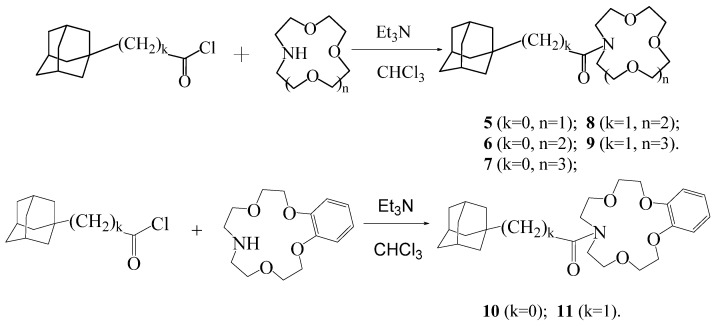
General method for the synthesis of macrocyclic adamantylamides **5**–**11**.

**Scheme 3:**
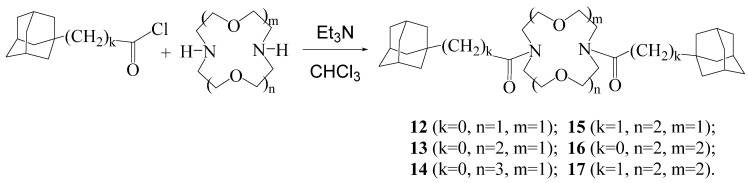
General method for the synthesis of diacyl derivatives **12**–**17**.

**Scheme 4:**
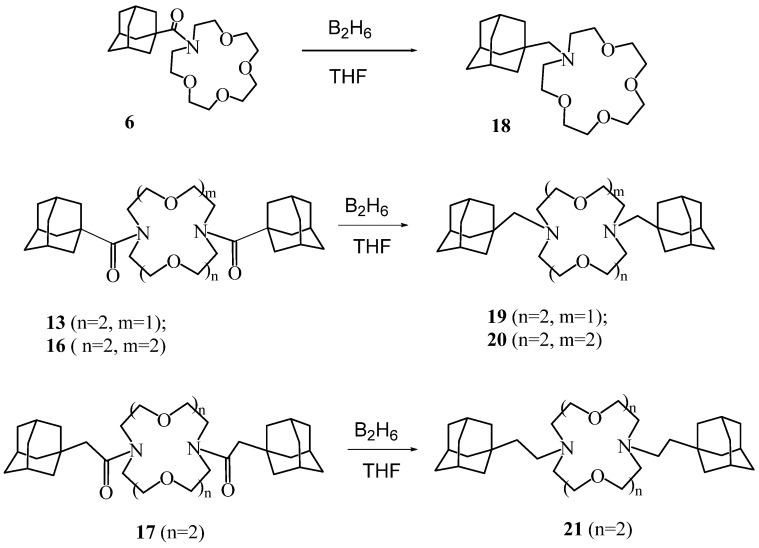
General method for the synthesis of adamantylalkyl derivatives **18**–**21**.

**Scheme 5:**
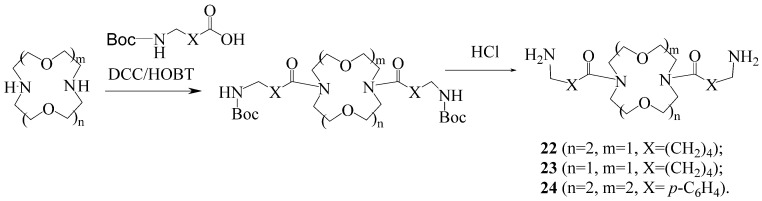
General method for the synthesis of diacyl derivatives **22**–**24**.

**Scheme 6:**
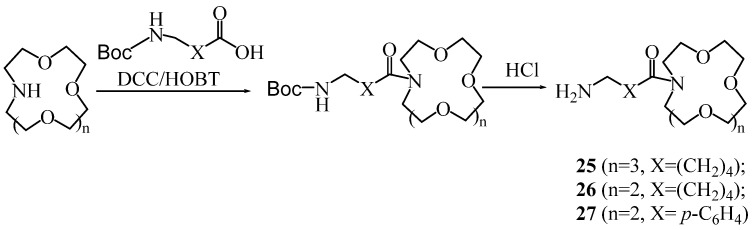
General method for the synthesis of monoacyl derivatives **25**–**27**.

**Scheme 7:**
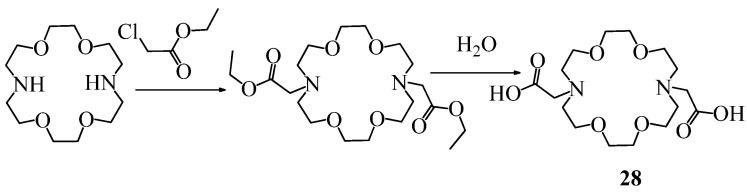
Synthesis of *N*,*N*’-*bis*(carboxymethyl)-4,13-diaza-18-crown-6 (**28**).

The investigated compounds were synthesized according to Scheme 1, Scheme 2, Scheme 3, Scheme 4, Scheme 5, Scheme 6 and Scheme 7. Adamantylamides **1**–**4** were synthesized by acylation of the corresponding amines with adamantane-t-carbonyl chloride in anhydrous chloroform in the presence of trimethylamine (Et_3_N) (Scheme 1).

Macrocyclic adamantylamides **5**–**11** were synthesized by acylation of the corresponding monoaza-crown ether with adamantane-1-carbonyl chloride or 2-(adamantan-1-yl)acetyl chloride in anhydrous chloroform in the presence of triethylamine (Scheme 2).

Derivatives of diaza-crown ethers **12**–**17** were obtained by acylation of amino groups with adamantane-1-carbonyl chloride or 2-(adamantan-1-yl)acetyl chloride in anhydrous chloroform in the presence of triethylamine (Scheme 3).

Adamantylalkyl derivatives of diaza-crown ethers **18**–**21** were obtained by reduction of previously synthesized macrocyclic amides **6**, **13**, **16**, **17** with diborane in tetrahydrofuran (THF) (Scheme 4).

Diacyl derivatives of diaza-crown ethers **22**–**24** were obtained by the carbodiimide method in the presence of hydroxybenzotriazole (HOBT), followed by removal of the *tert*-butoxycarbonyl (Boc) protecting group with HCl (Scheme 5).

Monoacyl derivatives **25**–**27** of aza-crown ethers were synthesized in a similar way (Scheme 6).

*N*,*N*′-*bis*(Carboxymethyl)-4,13-diaza-18-crown-6 (**28**) was obtained by alkylation of 4,13-diaza-18-crown-6 with chloroacetic acid ethyl ester in the presence of triethylamine, followed by hydrolysis [34] (Scheme 7).

### 2.2. Biological Activity of Aza-Crown Ethers

**Figure 1:**
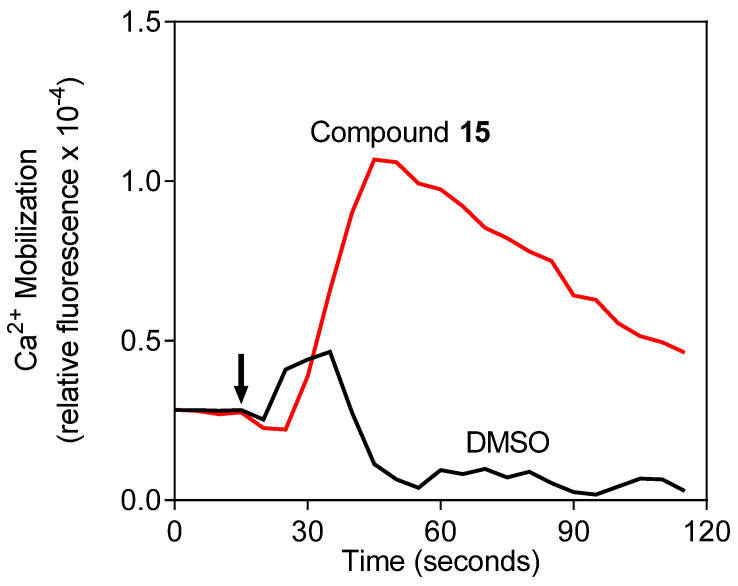
Effect of compound **15** on [Ca^2+^]_i_ in human neutrophils. Human neutrophils were treated with 5 µM of compound **15** or 1% dimethyl sulfoxide (DMSO) (negative control) and [Ca^2+^]_i_ was monitored (arrow indicates addition of treatment). Three independent experiments were performed, and all had similar results. The data shown are from one of these experiments.

We screened 24 aza- and diaza-crown ethers with adamantyl, adamantylalkyl, aminomethylbenzoyl, and ε-aminocaproyl substituents for their Ca^2+^ ionophore effects in human neutrophils. To monitor cytoplasmic Ca^2+^ levels, we used the fluorescent Ca^2+^ indicator Fluo-4, which is a dynamic single-wavelength fluorescent Ca^2+^ indicator. Increases in fluorescence intensity (λ_ex_ = 485 nm, λ_em_ = 538 nm) reflect the rise in cytoplasmic Ca^2+^ concentrations ([Ca^2+^]_i_; see Materials and Methods). We found that treatment with ten of the compounds (**8**, **10**–**17**, and **21**) elevated [Ca^2+^]_i_ in human neutrophils, with the most potent being compound **15** (*N*,*N*’-*bis*[2-(1-adamantyl)acetyl]-4,10-diaza-15-crown-5), which had an EC_50_ of 4.7 μM (Table 1). Compound **15** had very high efficacy, inducing a response similar in amplitude to that induced by the neutrophil agonist *f*MLF, while compounds **8**, **10**–**14**, **16**, **17**, and **21** were less effective (25–50% of the response induced by 5 nM *f*MLF). A representative time course for the elevation of neutrophil [Ca^2+^]_i_ by compound **15** is shown in Figure 1.

Although compounds **16**, **17**, **20**, **21**, **24**, and **28** all have diaza-18-crown-6 structures, only **16**, **17**, and **21** were active. Similarly, compounds **13**, **15**, **19**, and **22** all have diaza-15-crown-5 structures, whereas only **13** and **15** were active. Thus, it is evident from these data that the nature of additional substituents can affect Ca^2+^ binding and (or) transportation through cell membranes.

**Figure 2:**
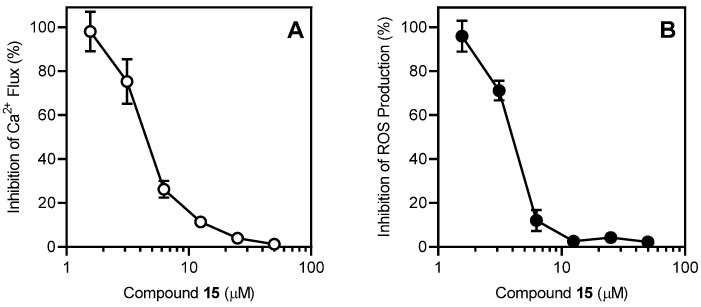
Inhibitory effect of compound **15** on Ca^2+^ mobilization and ROS production in human neutrophils. (**A**) Human neutrophils were treated with the indicated concentrations of compound **15** or 1% DMSO (negative control) for 10 min. The cells were activated by 5 nM *f*MLF, and [Ca^2+^]_i_ was monitored, as described. (**B**) Human neutrophils were treated with the indicated concentrations of compound **15** or 1% DMSO (negative control) for 10 min. The cells were activated by 200 nM PMA, and ROS production was monitored, as described. For each panel, the data are presented as the mean ± S.D. (N = 3) from one experiment that is representative of three independent experiments.

Since these compounds elevated neutrophil [Ca^2+^]_i_, and cytosolic Ca^2+^ flux is a key component of neutrophil activation [35,36], we evaluated if they could alter neutrophil activation by FPR1 or FPR2 agonists. Neutrophils and FPR-transfected HL60 cells were preincubated for 10 min with crown ether compounds, and [Ca^2+^]_i_ flux was stimulated with either an FPR1 agonist (*f*MLF for human neutrophils and FPR1-HL60 cells) or an FPR2 agonist (WKYMVM for FPR2-HL60 cells). We found that compounds **8**, **10**, **13**–**17**, and **21** inhibited the *f*MLF-induced increase in [Ca^2+^]_i_ in human neutrophils (Table 2), and a representative concentration-dependent response for the inhibition of the *f*MLF-induced increase in neutrophil [Ca^2+^]_i_ by compound **15** is shown in Figure 2A.

Compounds **8**, **10, 13**–**17**, and **21** also inhibited the *f*MLF-induced increase in [Ca^2+^]_i_ in FPR1-HL60 cells and the WKYMVM-induced increase in [Ca^2+^]_i_ in FPR2-HL60 cells (Table 1), indicating that these compounds disrupted Ca^2+^ mobilization induced by these G protein-coupled receptor (GPCR) peptide agonists. Note that three of the compounds that inhibited neutrophil [Ca^2+^]_i_ flux induced by the peptide agonist (see Table 1) had much higher IC_50_ values and targeted only one FPR subtype in transfected HL60 cells (compounds **8** and **11**) or had no effect at either receptor (compound **12**). It is not clear why FPR-transfected HL60 cells responded differently to these compounds. It is possible that this is due to differences in neutrophils and HL60 cells and will require further investigation.

To further investigate the effects of the most active crown ethers (those with IC_50_ < 10 µM), we analyzed the effects of compounds **8** and **11**–**17** on neutrophil chemotaxis. All eight of these compounds inhibited *f*MLF-induced neutrophil chemotaxis, with IC_50_ values ranging from 5 to 16 µM (Table 3), indicating that their effect on cytosolic Ca^2+^ was sufficient to inhibit neutrophil activation.

The most potent and receptor-specific FPR1 ligands described so far are the macrocyclic peptides, cyclosporines A and H [37]. Thus, we evaluated if our active macrocyclic aza-crown compounds might be binding to and blocking FPR1. The three most potent compounds (**13**–**15**) were analyzed for their ability to compete with WKYMVm-FITC for binding to FPR1, as described previously [38]. However, none of these compounds competed with WKYMVm-FITC and thus likely do not bind directly to FPR1.

To evaluate if the crown ethers could also disrupt GPCR-independent functional activity, we evaluated the effects of these compounds on phorbol 12-myristate 13-acetate (PMA)-stimulated reactive oxygen species (ROS) production. We found that compounds **13**–**15**, **17**, and **21** inhibited ROS production in human neutrophils, with the most potent being compound **15**, which had an IC_50_ of 4.1 µM (Table 2). A representative concentration-dependent response for the inhibition of PMA-stimulated neutrophil ROS production by compound **15** is shown in Figure 2B. Since ROS production in neutrophils also requires increased [Ca^2+^]_i_ [39,40], the results again confirm that the impact of these compounds on Ca^2+^ flux kinetics can alter neutrophil function.

To ensure that the results on inhibitory activity of the crown ethers were not due to possible compound toxicity, cytotoxicity of the active compounds was evaluated at various concentrations up to 25 µM in wild-type HL60 cells during a 90-min incubation period. None of the tested compounds affected cell viability at the highest tested concentrations (data not shown), thereby verifying that these compounds were not cytotoxic, at least during this relatively short incubation period.

Thus, our biological data indicate that the size of the macrocycle (compare compounds **8** and **9**) and the structure of the side chains (compare compounds **13** and **19**) both influence biological activity of the crown ethers. To characterize molecular conformations and electronic parameters of selected compounds, we performed molecular modeling.

### 2.3. Molecular Modeling

**Figure 3:**
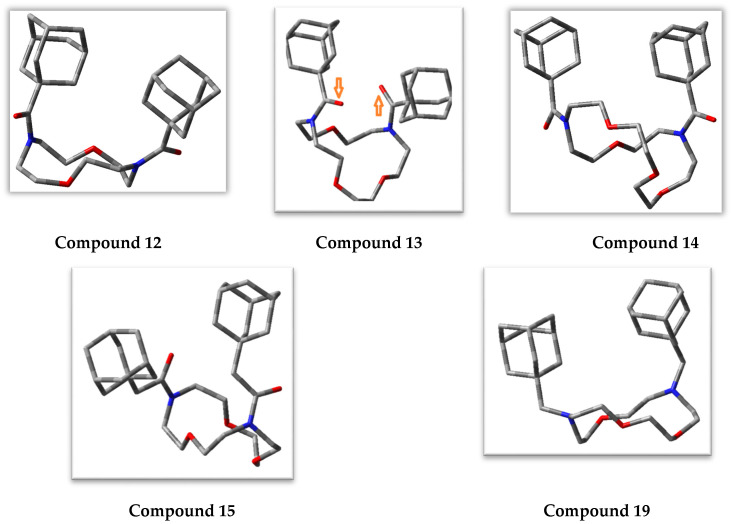
The low-energy conformers of compounds **12**–**15**, and **19**, according to density-functional theory (DFT) calculations with the BLYP functional. Hydrogen atoms are not shown. Carbonyl oxygen atoms in *anti*-orientation for the conformer of compound **13** are indicated with orange arrows.

Several crown ethers have been reported to be ionophores with various selectivity for alkali metal cations [17,41,42]. We performed density-functional theory (DFT) modeling of the interaction between selected diaza-crown ethers and several cations (Na^+^, K^+^, Ca^2+^) by evaluating their geometries and binding energies. Compounds **12**–**15**, and **19**, were chosen for molecular modeling in order to consider the effects of differences in ring sizes, the presence/absence of C=O groups, and differences in CH_2_ linkers attached to the adamantane moiety. To correctly calculate the energy of complex formation, it was necessary to perform a thorough conformational analysis of these macrocyclic ligands. We performed a systematic conformational search using molecular mechanics with subsequent optimization of the selected conformers by the semiempirical PM3 method. In addition, we employed a more accurate DFT method for individual low-energy conformers to find the optimal geometry for each of the aza-crown ethers. As shown in Figure 3, all heteroatoms of macrocycles **12**–**15**, together with both carbonyl oxygen atoms, formed a cavity that could potentially be capable of capturing positively charged ions. In the *anti*-orientation, one of the carbonyl oxygen atoms of molecule **13** is directed towards the shorter segment of the macrocycle, and the other one is oriented towards the longer segment (*anti*-oriented carbonyl groups are shown by arrows in Figure 3).

The conformation in which both carbonyl oxygen atoms of **13** are *syn*-oriented toward the shorter segment was also characterized by a favorable arrangement of all seven heteroatoms for cation capture. This conformation had a higher energy and was not present among the 30 pre-optimal conformations initially selected by molecular mechanics. Nevertheless, the possibility of complex formation with a ligand having the aforementioned *syn*-orientation of carbonyl groups was also considered. Similarly, the possibility of metal ion complexation with the compound containing these groups in *anti*-and *syn*-orientations was considered for diaza-crown ethers **12**, **14** (*anti*-oriented carbonyl groups in the lowest-energy conformation), and **15** (the most stable conformer with *syn*-oriented carbonyl groups) (Figure 3). Additionally, we considered the possibility of adopting a *trans*-orientation for the two adamantyl-containing substituents with their locations on opposite sides of the median plane of the macrocycle in the flexible ligand **14**.

**Figure 4:**
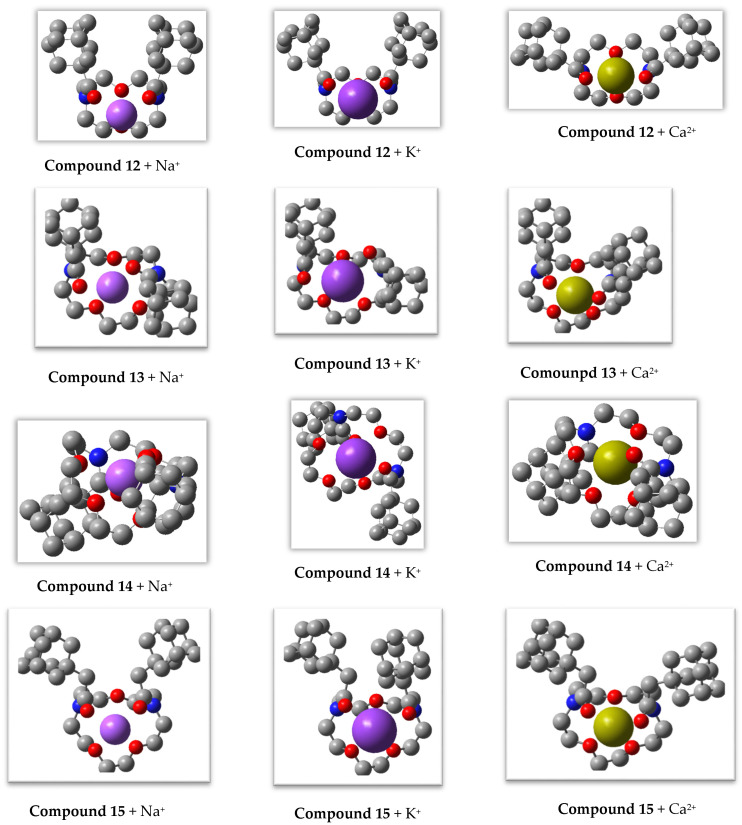

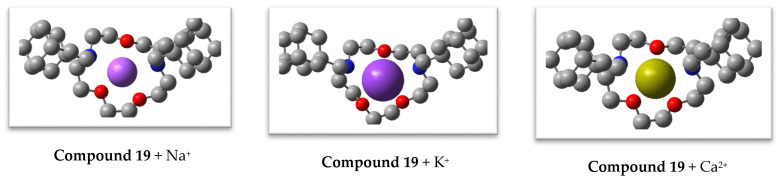
DFT-optimized geometric structures of Na^+^, K^+^, and Ca^2+^ complexes with **12**–**15**, and **19**, ligands. Hydrogen atoms are not shown.

Na^+^, K^+^, and Ca^2+^ ions were introduced into the central part of the conformer of compound **19**, as well as into the *syn*- and *anti*-conformers of **12**–**15** (for ligand **14** also into the *trans*-conformation), and the structures of the complexes were optimized by DFT modeling in the same approximation that was used for the diaza-crown ethers. The optimal geometric structures of the complexes are shown in Figure 4.

DFT analysis of these structures demonstrated that the complexes of all three cations with diaza-12-crown-4 ether **12** and diaza-15-crown-5 ether **15**, as well as of Ca^2+^ with diaza-15-crown-5 ether **13**, were characterized by a *syn*-arrangement of the carbonyl groups, while complexes of Na^+^ and K^+^ with ligand **13** had *anti*-oriented carbonyl groups. In complexes of diaza-18-crown-6 ether **14**, the ligand had a *trans*-arrangement of the adamantyl-containing substituents, with the two carbonyl oxygen atoms attached to a cation from opposite sides of the median plane formed by the four in-cycle oxygen atoms (Figure 4). Such a mode of chelation becomes possible because of the large size of the macrocycle in diaza-18-crown-6 ether **14**. The central cations were approximately “uniformly” surrounded by all oxygen atoms of ligands **13**, **14**, and diaza-15-crown-5 ether **19** (including the carbonyl oxygen atoms of **13** and **14**), and the metal-oxygen internuclear distances varied within rather narrow ranges (Table 4). The same is true for the Ca^2+^ complex with diaza-12-crown-4 ether **12**. In complexes with diaza-15-crown-5 ether **15**, four out of five oxygen atoms were relatively equidistant from a central cation, while either oxygen in the shorter segment connecting nitrogen atoms of the macrocycle was at a noticeably greater distance from a cation, especially in the case of alkali metal complexes (Table 4). Additionally, one of the macrocycle oxygen atoms in the Na^+^ and K^+^ complexes with ligand **12** was located 4.434 and 4.916 Å apart from the central cation, respectively. Nitrogen atoms were involved only in the complexation of ligand **19** with the cations. The metal-nitrogen distances in complexes with this diaza-crown ether were much shorter than those in the coordination compounds of the corresponding cations with ligands **12**–**14**, and **19**, which also contain amide functions. The positions of ions surrounded by heteroatoms within the complexes were fairly stable, and our attempts to displace ions from the positions within 1 Å led again to the structures shown in Figure 4 after further DFT optimization.

The charges on alkali metal ions bound with the investigated ligands were calculated in the approximation of natural bond orbitals. These charges were about 0.1 e lower than the formal charges of the cations. For the doubly charged Ca^2+^, this decrease was 0.18...0.21 e (Table 4). The exceptions were when Na^+^ and K^+^ ions were bound with diaza-crown ethers **12** and **15**, where the charges on the cations in these complexes were lower than formal values by only 0.021...0.049 e. The low degree of electron transfer from the ligand to cation in these cases is due to the remoteness of one of the oxygen atoms from the central ion (Table 4) and to the relatively high radius of K^+^. In addition, due to its large size, K^+^ does not completely fit inside the cavity of compound **19** and is positioned above the median plane of the macrocycle, unlike the Na^+^ ion (Figure 4). Therefore, the decrease in the formal charge of K^+^ in the complex with diaza-crown ether **19** is also small and equal to 0.056 e.

We calculated the binding energy ΔU for the complexation of Na^+^, K^+^, and Ca^2+^ ions with diaza-crown ethers according to the scheme:

M^n+^ + ligand → M^n+^·ligand, ΔU

As shown in Table 4, the ΔU values for the formation of Ca^2+^ complexes with ligands **12**–**15**, and **19** were significantly higher in absolute values than the binding energies for Na^+^ and K^+^. These results indicate a pronounced selectivity of the macrocyclic compounds for coordination with Ca^2+^. In contrast, aza-crown ethers containing fused phenazine fragments were reported to bind alkali metal ions somewhat better [43]. Thus, DFT calculations in gas phase for phenazine-containing ligands led to absolute values of ΔU in the range of 96.6–104.9 kcal/mol upon interaction with Na^+^ and K^+^, while the binding energy for Ca^2+^ did not exceed 79.0–88.9 kcal/mol [43]. In terms of ΔU, the coordination compounds of Ca^2+^ with the ligands reported here are among the most stable complexes of doubly charged cations with crown and aza-crown ethers [43,44]. The DFT results do not include solvation/desolvation effects on complex formation, which are very difficult to account for in calculations of charged species, especially for individual metal ions. Thus, the ΔU values obtained here refer to hypothetical gas phase processes, which is analogous to other reports [43,44,45,46].

**Figure 5:**
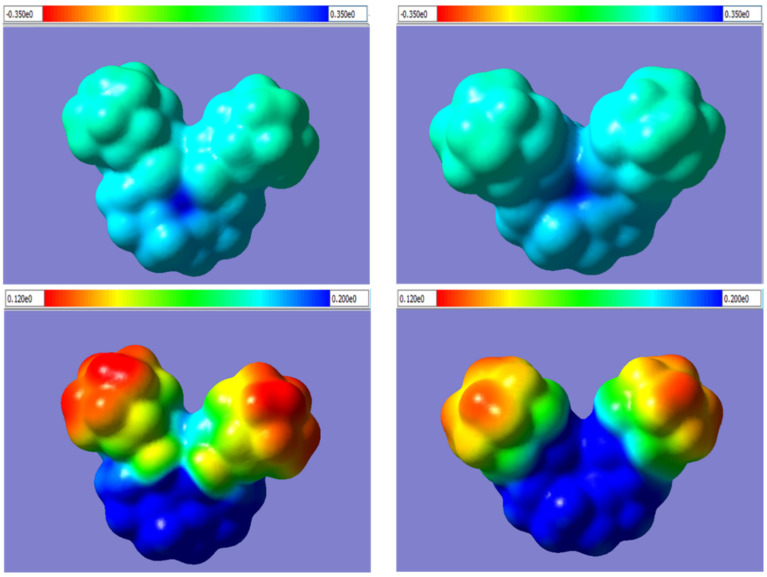
Electrostatic potential (ESP) mapped onto the isosurfaces of electron density (isovalue 0.0004) for Ca^2+^ complexes with diaza-crown ethers **15** (left panels) and **19** (right panels) calculated by the DFT method. Color shades correspond to different values of ESP, which vary from −0.35 (red) to 0.35 (blue) (upper panels) and from 0.12 and lower (red) to 0.20 and higher (dark-blue) (bottom panels).

The good geometric correspondence of Ca^2+^ to the cavity formed by the five oxygen atoms of ligands **12**–**15** (Figure 4, Table 4) indicates Ca^2+^ binds more effectively than alkali metal ions. This may be one of the reasons for the Ca^2+^-selective properties of these compounds in contrast to “classical” crown ethers like 18-crown-6 [7]. Complexation with aza- and diaza-crown ethers may help to explain the ionophore mechanism of Ca^2+^ transport. The bound ions are “hidden” inside the complex together with the polar nitrogen and oxygen atoms. “Outside” of these adducts are only non-polar and lipophilic adamantane substituents, as well as hydrocarbon groups of the macrocycles. This is illustrated in Figure 5, where shades of color indicate electrostatic potential (ESP) values mapped onto the electron density isosurfaces of the bound cations. Small dark-blue spots in the center of each upper panel correspond to highly positively charged areas occupied by Ca^2+^, which are surrounded by large and significantly less positively charged (light-blue) outer surfaces of macrocyclic ligands with the chosen ESP boundaries of the palette between −0.35 (highly negative, red) to 0.35 (highly positive, dark-blue). Assigning the ESP palette between 0.12 (low positive, red) to 0.2 (medium positive, dark blue) allows one to observe substantial differences in charge distributions in Ca^2+^ complexes with the carbonyl-containing compound **15** and the aza-crown ether **19** containing tertiary amine moieties. Indeed, the complex with **19** has smaller red and yellow areas of low positive ESP, which are prone to hydrophobic interactions, than the complex with aza-crown ether **15**. These differences may be one of the reasons for lower biological activity of aza-crown ether **19** in spite of its higher binding energy with Ca^2+^ and are in agreement with ionophore mechanism of biological action when the ability of a ligand to transfer cations through cell membranes is more important than strong binding with the cation.

We also performed quantitative structure-activity relationship (QSAR) analysis of the series of compounds **5**–**21** based on absorption, distribution, metabolism and excretion (ADME) parameters generated using the SwissADME web server [47]. The chosen subset contains all active compounds and several inactive aza-crown ethers. QSAR analysis of biologically-active crown- and aza-crown ethers was also previously performed by Supek et al. [16], where importance of the ligand physico-chemical properties was established. Here, we initially considered the following variables for the analysis: molecular weight (MW), number of heavy atoms (N_ha_), number of aromatic heavy atoms (N_ar_), fraction of sp^3^-carbons among all carbon atoms (C_sp3_), number of rotatable bonds (N_rot_), number of H-bond acceptors (N_HBA_), molar refractivity (MR), topological polar surface area (tPSA), measure of lipophilicity iLog P [48], number of carbonyl groups (N_C=O_), and number of adamantyl groups (N_Ad_). However, we found that MR, MW, and N_ha_ were tightly correlated with each other for our compounds, with pairwise correlation coefficients (r) within 0.99…1.00. Hence, from these three characteristics, we chose only MR as a representative independent variable for inclusion in the analysis. In addition, N_ar_ and C_sp3_ were correlated (r = −0.96). We constructed two QSAR models with inclusion of one of these two variables along with N_rot_, N_HBA_, MR, N_C=O_, N_ad_, tPSA, and iLog P (Table 5). To account for inactive compounds, we used the reciprocal values of EC_50_ in human neutrophils as a measure of biological activity (elevation of neutrophil [Ca^2+^]_i_), where inactive compounds were assigned a value of zero. This measure (1/[EC_50_]) was used as a dependent variable.

Forward stepwise regression analyses with the data presented in Table 5 and alternate inclusion of N_ar_ or C_sp3_ gave the best regression Equations (1) and (2) with four variables:

1/EC_50_ = −0.058 + 0.230∙N_ar_ − 0.574 tPSA + 0.471 iLog P + 0.797∙N_C=O_; n = 24, R = 0.778, s = 0.040 (1)

1/EC_50_ = 0.056 − 0.281∙Csp^3^ − 0.588 tPSA + 0.518 iLog P + 0.763∙N_C=O_; n = 24, R = 0.784, s = 0.039 (2)

**Figure 6:**
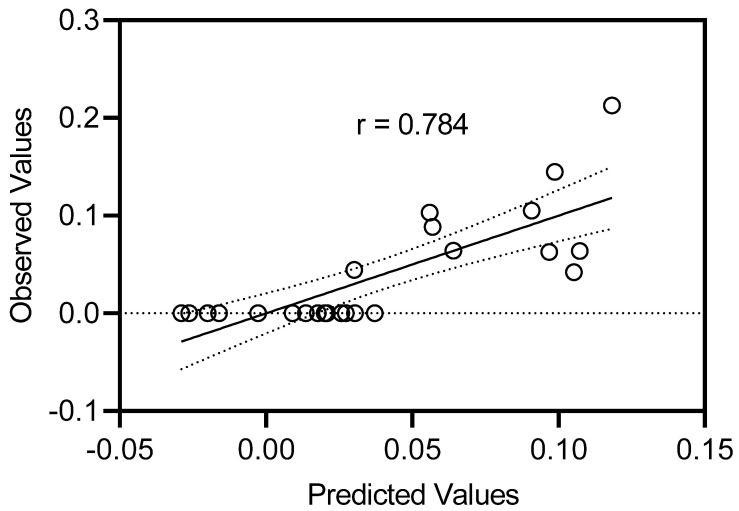
Plot of calculated vs. experimental biological activities (1/EC_50_) obtained with regression Equation (2).

The plot of calculated vs. experimental biological activities for the best QSAR model (2) is shown in Figure 6.

The regressions were of comparable quality with multiple correlation coefficients of ~0.784. Both models include iLog P and N_C=O_ with positive coefficients and tPSA with a negative coefficient, i.e., the presence of carbonyl groups and an increase of hydrophobicity favor biological activity. According to Equations (1) and (2), the presence of benzene rings or a corresponding decrease of C_sp3_ were also favorable for biological activity. Aromatic rings are lipophilic moieties that enhance shielding of the positively charged central ions from the hydrophobic environment during cation transportation. The DFT-optimized structures of the complexes showed that the carbonyl oxygen atoms of the aza-crown ethers participated in cation binding (Figure 4). Thus, they can play an important role in Ca^2+^ transfer through cell membranes. It should be noted that the previously reported U-shaped relationship between biological activity and lipophilicity [16] was not observed for the series of compounds shown here.

Our results indicate that ADME characteristics of the ligands largely define their Ca^2+^ ionophore properties. According to the DFT results, Ca^2+^ can be more effectively bound by the diaza-crown ethers than Na^+^ or K^+^. Noticeably, the binding energies ΔU of the Ca^2+^ complexes (Table 3) did not correlate with the observed biological activities of diaza-crown ethers investigated by the DFT method. Clearly, there is a need not only for strong binding of the cation, but also for its relatively easy ion transport through membranes and release at the final stage of transport for good ionophore properties of the macrocyclic ligands, as discussed above (Figure 5).

## 3. Materials and Methods

### 3.1. General Information

The structures of the synthesized compounds were determined by ^1^H and ^13^C NMR on a Bruker AVANCE DRX 500 spectrometer operating at 499.87 MHz (^1^H) and 125.69 MHz (^13^C) for ~10% solutions in dimethyl sulfoxide (DMSO)-d_6_ and CDCl_3_ with tetramethylsilane (TMS) as an internal standard. Mass spectra were obtained by the fast atom bombardment (FAB) method on a VG 70-70EQ mass spectrometer using an 8 kV Xe atomic beam and *m*-nitrobenzyl alcohol as a matrix. Melting points were measured in open capillaries and are not corrected. The reactions were controlled by TLC on Kieselgel 60 F254 plates (Merck); purification was carried out by crystallization or column chromatography on silica gel (Kieselgel 60 0.063–0.100 mm, Merk). Purity of the compounds was determined by high-performance liquid chromatograph (HPLC) using a Shimadzu chromatography system (controller of the CBM-20A system; vacuum degasser DGU-20 A5; high-pressure pump LC-20AD UFLC equipped with a 4-channel low-pressure gradient block; column thermostat CTO-20A; diode matrix detector SPD-M20A) equipped with an Acclaim Polar Advantage II 3 μm column (4.6 mm × 150 mm) with a Guard cartridge and a manual injector with a 10 μL loop. Mobile phase: acetonitrile (30%) and 0.1% solution of trifluoroacetic acid in deionized water (70%). Mobile phase flow rate: 1.0 mL/min. The injection volume was 10 μL. Thermostat temperature: 20 °C. UV scanning range: 190–400 nm. The purity of the synthesized compounds was at least 98.5% according to HPLC.

The starting aza-crown ethers (aza-15-crown-5, aza-18-crown-6, benzoaza-15-crown-5, 4,10-diaza-12-crown-4, 4,10-diaza-15-crown-5, 4,10-diaza-18-crown-6) [49,50,51], 6-(Boc-amino)hexanoic and 4-(Boc-aminomethyl)benzoic acids, adamantane-1-carbonyl chloride, 2-(adamantan-1-yl)acetyl chloride, and diborane [52,53] were obtained by standard methods. Their spectral characteristics and physical constants were identical to those given in the literature.

### 3.2. Chemistry

#### 3.2.1. General Procedure for Synthesis Compounds **1**–**4**, **10**, and **11**

To a solution of 10 mmol of amine or the corresponding monoaza-crown ether in 10 mL of anhydrous chloroform, triethylamine 1.53 mL (11 mmol) was added. The reaction mixture was cooled to 0 °C, and a solution of 10 mmol adamantane-1-carbonyl chloride or 2-(adamantan-1-yl)acetyl chloride in 10 mL of anhydrous chloroform was added dropwise for 5 min with vigorous stirring. The reaction mixture was stirred for 2 h, and 40 mL of chloroform was added. The combined organic phase was washed successively with water (2 × 10 mL), 1 N hydrochloric acid (1 × 10 mL), H_2_O (1 × 1 mL), 10% Na_2_CO_3_ solution (2 × 10 mL), and H_2_O (1 × 10 mL). The organic phase was dried with anhydrous Na_2_SO_4_, and the solvent was removed on a rotary evaporator to dryness. The products were purified by recrystallization from hexane.

*Methyl 6-(adamantane-1-carboxamido)hexanoate* (**1**). Yield 85%. Colorless crystals. MP 52–56 °C. NMR ^1^H (CDCl_3_) δ_H_, 1.28–1.34 (m, 2H, CH_2_), 1.45–1.51 (m, 2H, CH_2_), 1.59–1.63 (m, 2H, CH_2_), 1.65–1.73 (m, 6H, Ad), 1.82 (br.s, 6H, Ad), 2.01 (br.s, 3H, Ad), 2.28–2.31 (m, 2H, CH_2_), 3.19–3.23 (m, 2H, CH_2_), 3.64 (m, 3H, CH_3_), 5.67 (br.s, 1H, NH). NMR ^13^C (CDCl_3_) δ_C_, 24.46 (1C, CH_2_), 26.29 (1C, CH_2_), 28.14 (3C, Ad), 29.26 (1C, CH_2_), 33.85 (1C, CH_2_), 36.52 (3C, Ad), 38.97 (1C, CH_2_), 39.27 (3C, Ad), 40.55 (1C, Ad), 51.47 (1C, CH_3_), 174.04 (1C, COO), 177.97(1C, CON). MS, *m/z*: 292 (M+H)^+^.

*N-(1-adamantyl)adamantane-1-carboxamide* (**2**). Yield 87%. Colorless crystals. MP 255–257 °C. NMR ^1^H (CDCl_3_) δ_H_, 1.67–1.74 (m, 12H, Ad), 1.80 (br.s, 6H, Ad), 1.98 (br.s, 6H, Ad), 2.02 (br.s, 3H, Ad), 2.06 (br.s, 3H, Ad), 5.22 (br.s, 1H, NH). NMR ^13^C (CDCl_3_) δ_C_, 28.26 (3C, Ad), 29.50 (3C, Ad), 36.44 (3C, Ad), 36.58 (3C, Ad), 39.42 (3C, Ad), 40.88 (1C, AdCO), 41.66 (3C, Ad), 51.18 (1C, AdNH), 177.23 (1C, CON). MS, *m/z*: 314 (M+H)^+^.

*N,2-di(1-adamantyl)acetamide* (**3**). Yield 93%. Colorless crystals. MP 242–244 °C. NMR ^1^H (CDCl_3_) δ_H_, 1.46 (br.s, 6H, Ad), 1.61–1.64 (m, 3H, Ad), 1.70–1.79 (m, 9H, Ad), 1.87 (br.s, 6H, Ad), 1.97 (br.s, 3H, Ad), 2.04 (br.s, 3H, Ad), 2.93–2.95 (d, *J* 6.2 Hz, 2H, Ad), 5.63 (br.s, 1H, NH). NMR ^13^C (CDCl_3_) δ_C_, 28.22 (3C, Ad), 28.24 (3C, Ad), 33.82 (1C, CH_2_), 36.58 (3C, Ad), 36.99 (3C, Ad), 39.52 (3C, Ad), 40.32 (3C, Ad), 40.88 (1C, Ad), 50.42 (1C, AdNH), 177.87 (1C, CON). MS, *m/z*: 328 (M+H)^+^.

*N-cyclohexyladamantane-1-carboxamide* (**4**). Yield 96%. Colorless crystals. MP 174–176 °C. NMR ^1^H (CDCl_3_) δ_H_, 1.06–1.20 (m, 3H, CH_2_), 1.33–1.40 (m, 2H, CH_2_), 1.59–1.63 (m, 1H, CH_2_), 1.68–1.75 (m, 6H, Ad, 2H, CH_2_), 1.83 (br.s, 6H, Ad), 1.86–1.88 (m, 2H, CH_2_), 2.03 (br.s, 3H, Ad), 3.72–3.78 (m, 1H, CH), 5.41 (br.s, 1H, NH). NMR ^13^C (CDCl_3_) δ_C_, 24.86 (2C, CH_2_), 25.62 (1C, CH_2_), 28.19 (3C, Ad), 33.19 (2C, CH_2_), 36.57 (3C, Ad), 39.30 (3C, Ad), 40.43 (1C, Ad), 47.56 (1C, CH_2_), 176.96 (1C, CON). MS, *m/z*: 262 (M+H)^+^.

*N-[1-Oxo-1-(1-adamantyl)methyl]benzoaza-15-crown-5* (**10**). Yield 89%. Colorless crystals. MP 111–113 °C. NMR ^1^H (CDCl_3_) δ_H_, 1.72 (br.s, 6H, Ad), 2.00 (br.s, 6H, Ad), 2.03 (br.s, 3H, Ad), 3.67 (br.s, 4H, CH_2_N), 3.87 (br.s, 8H, CH_2_O), 4.11 (br.s, 4H, CH_2_O), 6.90 (d, *J* 12.0 Hz, 4H, Ar). NMR ^13^C (CDCl_3_) δ_C_, 28.55 (3C, Ad), 36.62 (3C, Ad), 39.11 (3C, Ad), 42.16 (1C, Ad), 50.68 (2C, CH_2_N), 69.38 (2C, CH_2_O), 69.86 (2C, CH_2_O), 71.07 (2C, CH_2_O), 113.30 (2C, Ar), 121.30 (2C, Ar), 148.80 (2C, Ar), 177.19 (1C, CON). MS, *m/z*: 430 (M+H)^+^.

*N-[2-(1-adamantyl)acetyl]benzoaza-15-crown-5* (**11**). Yield 90%. Colorless crystals. MP 141–142 °C. NMR ^1^H (CDCl_3_) δ_H_, 1.63–1.69 (m, 12H, Ad), 1.94 (br.s, 3H, Ad), 2.14 (br.s, 2H, CH_2_), 3.58–3.64 (m, 4H, CH_2_N), 3.72–3.74 (m, 2H, CH_2_O), 3.80–3.82 (m, 2H, CH_2_O), 3.93–3.98 (m, 4H, CH_2_O), 4.10–4.14 (m, 4H, CH_2_O), 6.87–6.93 (m, 4H, Ar). ^13^C NMR (CDCl_3_) δ_C_, 28.74 (3C, Ad), 33.68 (1C, Ad), 36.87 (3C, Ad), 42.72 (3C, Ad), 45.84 (1C, CH_2_Ad), 49.41 (1C, CH_2_N), 51.10 (1C, CH_2_N), 68.50 (1C, CH_2_O), 69.36 (1C, CH_2_O), 70.01 (1C, CH_2_O), 70.10 (1C, CH_2_O), 70.34 (1C, CH_2_O), 71.11 (1C, CH_2_O), 113.28 (1C, Ar), 113. 33 (1C, Ar), 121.28 (1C, Ar), 121.37 (1C, Ar), 148.72 (1C, Ar), 148.81 (1C, Ar), 171.85 (1C, CON). MS, *m/z*: 444 (M+H)^+^.

*N*-[1-Oxo-1-(1-adamantyl)methyl]aza-12-crown-4 (**5**), *N*-[1-oxo-1-(1-adamantyl)methyl]aza-15-crown-5 (**6**), *N*-[1-oxo-1-(1-adamantyl)methyl]aza-18-crown-6 (**7**), N-[2-(1-adamantyl)acetyl]aza-15-crown-5 (**8**), and *N*-[2-(1-adamantyl)acetyl]aza-18-crown-6 (**9**) were synthesized as described previously [17,54,55].

#### 3.2.2. General Procedure for Synthesis Compounds **14** and **15**

To a solution of 10 mol of the corresponding diaza-crown ether in 15 mL of anhydrous chloroform, triethylamine 3.5 mL (25 mmol) was added. The reaction mixture was cooled to 0 °C, and a solution of 21 mmol adamantane-1-carbonyl chloride or 2-(adamantan-1-yl)acetyl chloride in 15 mL of anhydrous chloroform was added dropwise for 5 min with vigorous stirring. The reaction mixture was stirred for 2 h, and 40 mL of chloroform was added. The combined organic phase was washed successively with H_2_O (2 × 10 mL), 1 N HCl (1 × 10 mL), water (1 × 10 mL), 10% Na_2_CO_3_ solution (2 × 10 mL), and H_2_O (1 × 10 mL). The organic phase was dried with anhydrous Na_2_SO_4_, and the solvent was removed on a rotary evaporator to dryness. The products were purified by recrystallization from hexane.

*N,N’-bis[1-oxo-1-(1-adamantyl)methyl]-4,10-diaza-18-crown-6* (**14**). Yield 97%. Colorless crystals. MP 91–93 °C. NMR ^1^H (CDCl_3_) δ_H_, 1.71 (br.s, 12H, Ad), 1.98 (br.s, 12H, Ad), 2.03 (br.s, 6H, Ad), 3.60–3.68 (m, 24H, CH_2_O). NMR ^13^C (CDCl_3_) δ_C_, 28.53 (6C, Ad), 36.61 (6C, Ad), 39.19 (6C, Ad), 42.09 (2C, Ad), 49.07 (2C, CH_2_N), 49.52 (2C, CH_2_N), 70.32 (4C, CH_2_O), 70.57 (2C, CH_2_O), 70.92 (2C, CH_2_O), 177.01 (2C, CON). MS, *m/z*: 531 (M+H)^+^.

*N,N’-bis[2-(1-adamantyl)acetyl]-4,10-diaza-15-crown-5* (**15**). Yield 91%. Colorless crystals. MP 72–76 °C. NMR ^1^H (CDCl_3_) δ_H_, 1.63–1.70 (m, 24H, Ad), 1.95 (br.s, 6H, Ad), 2.08–2.17 (m, 4H, CH_2_Ad), 3.55–3.73 (m, 20H, CH_2_O). NMR ^13^C (CDCl_3_) δ_C_, 28.73 (6C, Ad), 33.67 (2C, Ad), 36.85 (6C, Ad), 42.77 (6C, Ad), 45.95 (2C, CH_2_Ad), 48.59 (2C, CH_2_N), 50.30 (2C, CH_2_N), 69.04 (2C, CH_2_O), 70.23 (1C, CH_2_O), 70.55 (1C, CH_2_O), 70.74 (1C, CH_2_O), 71.04 (1C, CH_2_O), 172.02 (1C, CON). MS, *m/z*: 571 (M+H)^+^.

*N*,*N*’-bis[1-oxo-1-(1-adamantyl)methyl]-4,10-diaza-12-crown-4 (**12**), *N*,*N*’-bis[1-oxo-1-(1-adamantyl)methyl]-4,10-diaza-15-crown-5 (**13**), *N*,*N*’-bis[1-oxo-1-(1-adamantyl)methyl]-4,13-diaza-18-crown-6 (**16**), and *N*,*N*’-bis[2-(1-adamantyl)acetyl]-4,13-diaza-18-crown-6 (**17**) were synthesized as described previously [16,54].

#### 3.2.3. General Procedure for Synthesis Compound **19**

To a suspension of 15 mmol NaBH_4_ in 20 mL of anhydrous THF, a solution of 15 mmol boron trifluoride etherate in 20 mL of anhydrous THF was added dropwise with stirring. The temperature was brought to 50 °C, and the reaction mixture was stirred for 30 min and cooled. The precipitate formed was filtered under reduced pressure. To the resulting filtrate, a solution of 0.015 mol of **13** in 30 mL THF was added for 30 min at room temperature. The reaction mixture was stirred at reflux for 4 h and cooled. Next, 15 mL of a 10% HCl was added, and the reflux continued for additional 3 h. After cooling, the mixture was neutralized with concentrated NaOH solution, the pH was adjusted to 9–10, and the reaction products were extracted with CHCl_3_ (5 × 15 mL). The combined extracts were dried with MgSO_4_. After removal of CHCl_3_ under reduced pressure, the product (**19**) was recrystallized twice from anhydrous hexane.

*N,N’-bis[(1-adamantyl)methyl]-4,10-diaza-15-crown-5* (**19**). Yield 94%, light yellow crystals, MP 74–78 °C. NMR ^1^H (CDCl_3_) δ_H_, 1.47–1.50 (m, 12H, Ad), 1.60–1.63 (m, 6H, Ad), 1.68–1.70 (m, 6H, Ad), 1.93 (br.s, 6H, Ad), 2.11 (br.s, 4H, CH_2_), 2.72–2.77 (m, 8H, CH_2_N), 3.56–3.62 (m, 12H, CH_2_O). NMR ^13^C (CDCl_3_) δ_C_, 28.55 (6C, Ad), 35.10 (2C, Ad), 37.29 (6C, Ad), 41.14 (6C, Ad), 57.73 (2C, CH_2_N), 57.78 (2C, CH_2_N), 70.24 (2C, CH_2_O), 70.80 (2C, CH_2_), 70.96 (2C, CH_2_O), 71.48 (2C, CH_2_O). MS, *m/z*: 533 (M+H)^+^.

*N*-[(1-adamantyl)methyl]aza-15-crown-5 (**18**), *N*,*N*’-bis[(1-adamantyl)methyl]-4,13-diaza-18-crown-6 (**20**), and *N*,*N*’-bis[2-(1-adamantyl)ethyl]-4,13-diaza-18-crown-6 (**21**) were synthesized as described previously [16,54].

#### 3.2.4. General Procedure for Synthesis Compounds **22** and **23**

To a solution of 11 mmol of 6-(Boc-amino)hexanoic acid, 1.49 g (11 mmol) of 1-hydroxybenzotriazole in 5 mL of anhydrous dioxane and 15 mL of anhydrous dichloromethane, 2.27 g (11 mmol) of *N*,*N*’-dicyclohexylcarbodiimide were added at room temperature. The mixture was stirred for 1 h, and 5 mmol of the corresponding diaza-crown ether was added. The mixture was stirred for additional 4–5 h (the reaction was monitored by TLC using CHCl_3_:methanol:ammonia (25%), 5:3:1 as eluent). Precipitated dicyclohexylurea was filtered and the organic phase was washed successively with 0.05 N HCl (4 × 10 mL), H_2_O (10 mL), Na_2_CO_3_ solution (0.05 N) (4 × 10 mL), H_2_O (10 mL) and dried with anhydrous Na_2_SO_4_. The solvent was evaporated to dryness under reduced pressure. The crude product was purified by column chromatography using ethyl acetate: methanol (20:1) as eluent. To remove the Boc protection, 10 mL of 4 N HCl in ethyl acetate was added to a solution of 5 mmol of the corresponding Boc derivative in 5 mL of anhydrous ethyl acetate. The reaction mixture was stirred for 1 h, the solvent was removed to dryness on a rotary evaporator, and the product was dried under vacuum to constant weight.

*N,N’-bis(6-aminohexanoyl)-4,10-diaza-15-crown-5 dihydrochloride* (**22**). Yield 94%, light yellow crystals, MP 114–118 °C. NMR ^1^H (DMSO-d_6_) δ_H_, 1.25 (br.s, 4H, CH_2_), 1.36 (br.s, 4H, CH_2_), 1.46 (br.s., 4H, CH_2_), 2.25–2.29 (m, 4H, CH_2_), 2.56 (br.s, 2H, CH_2_), 2.88 (br.s, 2H, CH_2_), 3.45–3.61 (m, 20H, CH_2_O). NMR ^13^C (DMSO-d_6_) δ_C_, 25.12 (2C, CH_2_), 26.55 (2C, CH_2_), 32.75 (2C, CH_2_), 32.88 (2C, CH_2_), 40.43 (2C, CH_2_), 48.11 (2C, CH_2_N), 49.44 (2C, CH_2_N), 69.36 (2C, CH_2_O), 70.09 (2C, CH_2_O), 70.68 (2C, CH_2_O), 172.97 (2C, CON). MS, *m/z*: 445 (M+H)^+^.

*N,N’-bis(6-aminohexanoyl)-4,10-diaza-12-crown-4 dihydrochloride* (**23**). Yield 93%, light yellow oil. NMR ^1^H (DMSO-d_6_) δ_H_, 1.24 (br.s, 4H, CH), 1.36 (br.s, 4H, CH_2_), 1.45 (br.s, 4H, CH_2_), 2.27–2.29 (m, 4H, CH_2_), 2.56 (br.s, 2H, CH_2_), 2.87 (br.s, 2H, CH_2_), 3.33–3.35 (m, 4H, CH_2_O), 3.42–3.43 (m, 4H, CH_2_O), 3.49 (br.s, 2H, CH_2_O), 3.55 (br.s, 2H, CH_2_O), 3.60–3.63 (m, 4H, CH_2_O). NMR ^13^C (DMSO-d_6_) δ_C_, 24.98 (2C, CH_2_), 26.51 (2C, CH_2_), 26.66 (2C, CH_2_), 33.12 (2C, CH_2_), 40.83 (2C, CH_2_), 48.30 (1C, CH_2_N), 48.78 (1C, CH_2_N), 49.94 (1C, CH_2_N), 50.95 (1C, CH_2_N), 67.97 (1C, CH_2_O), 68.35 (1C, CH_2_O), 69.39 (1C, CH_2_O), 69.55 (1C, CH_2_O), 173.16 (2C, CON). MS, *m/z*: 401 (M+H)^+^.

*N,N’-bis[4-(aminomethyl)benzoyl]-4,13-diaza-18-crown-6 dihydrochloride* (**24**) was synthesized as described previously [56].

#### 3.2.5. General Procedure for Synthesis Compounds **25**–**27**

To a solution of 10.5 mmol of 6-(Boc-amino)hexanoic or 4-(Boc-aminomethyl)benzoic acid, 1.42 g (10.5 mmol) of 1-hydroxybenzotriazole in 5 mL of anhydrous dioxane and 15 mL of anhydrous dichloromethane, 2.17 g (10.5 mmol) *N*,*N*’-dicyclohexylcarbodiimide were added at room temperature. The mixture was stirred for 1 h, and 10 mmol of aza-15-crown-5 or aza-18-crown-6 was added. The mixture was stirred for an additional 4–5 h (the reaction was monitored by TLC using CHCl_3_:methanol:ammonia (25%) 5:3:1 as eluent). Precipitated dicyclohexylurea was filtered, and the organic phase was washed successively with 0.05 N HCl (4 × 10 mL), H_2_O (10 mL), Na_2_CO_3_ solution (0.05 N) (4 × 10 mL), H_2_O (10 mL) and dried with anhydrous Na_2_SO_4_. The solvent was removed to dryness under reduced pressure. The crude product was purified by column chromatography using ethyl acetate: methanol (20:1) as eluent. To remove the Boc protection, 15 mL of 4 M HCl in ethyl acetate was added to a solution of 3.3 mmol of the corresponding Boc derivative in 5 mL of anhydrous ethyl acetate. The reaction mixture was stirred for 1 h, the solvent was removed to dryness on a rotary evaporator, and the product was dried under vacuum to constant weight.

*N-(6-aminohexanoyl)aza-18-crown-6 hydrochloride* (**25**). Yield 96%, light yellow oil. NMR ^1^H (DMSO-d_6_) δ_H_, 1.28–1.31 (m, 2H, CH_2_), 1.46–1.49 (m, 2H, CH_2_), 1.54–1.57 (m, 2H, CH_2_), 2.29–2.31 (m, 2H, CH_2_), 3.38–3.52 (m, 26H, CH_2_O; CH_2_). NMR ^13^C (DMSO-d_6_) δ_C_, 24.75 (1C, CH_2_), 26.06 (1C, CH_2_), 27.28 (1C, CH_2_), 32.36 (1C, CH_2_), 39.06 (1C, CH_2_), 46.57 (2C, CH_2_N), 69.09 (2C, CH_2_O), 70.20 (2C, CH_2_O), 70.36 (2C, CH_2_O), 70.52 (2C, CH_2_O), 70.63 (2C, CH_2_O), 172.46 (C, CON). MS, *m/z*: 377 (M+H)^+^.

*N-(6-aminohexanoyl)aza-15-crown-5 hydrochloride* (**26**). Yield 90%, light yellow oil. NMR ^1^H (DMSO-d_6_) δ_H_, 1.28–1.31 (m, 2H, CH_2_), 1.46–1.50 (m, 2H, CH_2_), 1.54–1.58 (m, 2H, CH_2_), 2.27–2.30 (m, 2H, CH_2_), 3.00–3.10 (m, 2H CH_2_), 3.39–3.55 (m, 20H, CH_2_O). NMR ^13^C (DMSO-d_6_) δ_C_, 24.43 (1C, CH_2_), 25.86 (1C, CH_2_), 27.09 (1C, CH_2_), 32.47 (1C, CH_2_), 39.01 (1C, CH_2_), 49.58 (2C, CH_2_N), 66.02 (2C, CH_2_O), 69.85 (2C, CH_2_O), 70.21 (2C, CH_2_O), 70.69 (2C, CH_2O_O), 174.77 (1C, CON). MS, *m/z*: 333 (M+H)^+^.

*N-[4-(aminomethyl)benzoyl)aza-15-crown-5 hydrochloride* (**27**). Yield 91%, light yellow oil. NMR ^1^H (DMSO-d_6_) δ_H_, 3.40–3.56 (m, 20H, CH_2_O), 3.69–3.77 (m, 2H, ArCH_2_N), 7.38–7.40 (d, *J* 7.5 Hz, 2H, Ar), 7.55–7.57 (d, *J* 7.5 Hz, 2H, Ar). NMR ^13^C (DMSO-d_6_) δ_C_, 42.26 (1C, CH_2_NH_2_), 47.33 (2C, CH_2_N), 69.89 (2C, CH_2_O), 70.01 (2C, CH_2_O), 70.21 (2C, CH_2_O), 70.81 (2C, CH_2_O), 127.17 (2C, Ar), 130.31 (2C, Ar), 137.40 (1C, Ar), 140.09 (1C, Ar), 171.02 (1C, CON). MS, *m/z*: 353 (M+H)^+^.

*N,N’-bis(carboxymethyl)-4,13-diaza-18-crown-6* (**28**) was synthesized as described in paper [34].

### 3.3. Biological Methods

#### 3.3.1. Materials for Biological Studies

Dimethyl sulfoxide (DMSO), *N*-formyl Met-Leu-Phe (*f*MLF), phorbol 12-myristate 13-acetate (PMA), HEPES, and Histopaque 1077 were from Sigma Chemical Co. (St. Louis, MO, USA). RPMI 1640 medium and penicillin–streptomycin solution were from Mediatech (Herdon, VA, USA). Fetal bovine serum (FBS) was from Atlas Biologicals (Fort Collins, CO, USA). Peptide Trp-Lys-Tyr-Met-Val-Met (WKYMVM) was from Tocris Bioscience (Ellisville, MO, USA). Hanks’ balanced salt solution (HBSS), Fluo-4 AM, and G418 were from Life Technologies (Grand Island, NY, USA). HBSS containing 1.3 mM CaCl_2_ and 1.0 mM MgSO_4_ is designated as HBSS^+^; HBSS without ions Ca^2+^ and Mg^2+^ is designated as HBSS^−^. Fluorescein isothiocyanate (FITC) was conjugated to the lysine residue of Trp-Lys-Tyr-Met-Val-D-Met (WKYMVm) to produce a fluorescent ligand (WKYMVm-FITC) that binds to FPR1 (custom synthesis by Bachem, Torrance, CA, USA). All synthesized compounds for biological testing were dissolved in DMSO at 5 mM stock concentration and stored at −20 °C.

#### 3.3.2. Cell Culture

Human promyelocytic leukemia HL60 cells stably transfected with FPR1 (FPR1-HL60 cells) or FPR2 (FPR2-HL60 cells) (kind gifts from Dr. Marie-Josephe Rabiet, INSERM, Grenoble, France) were cultured in RPMI 1640 medium supplemented with 10% heat-inactivated fetal calf serum, 10 mM HEPES, 100 μg/mL streptomycin, 100 U/mL penicillin, and G418 (1 mg/mL). Although stable cell lines are cultured under G418 selection pressure, G418 may affect some assays, so it was removed in the last round of culture before assays were performed.

#### 3.3.3. Isolation of Human Neutrophils

For isolation of human neutrophils, blood was collected from healthy donors in accordance with a protocol approved by the Institutional Review Board at Montana State University. Neutrophils were purified from the blood using dextran sedimentation, followed by Histopaque 1077 gradient separation and hypotonic lysis of red blood cells, as described previously [57]. Isolated neutrophils were washed twice and resuspended in HBSS^−^. Neutrophil preparations were routinely >95% pure, as determined by light microscopy, and >98% viable, as determined by trypan blue exclusion. Neutrophils were obtained from multiple different donors; however, the cells from different donors were never pooled during experiments.

#### 3.3.4. Ca^2+^ Mobilization Assay

Changes in neutrophil intracellular Ca^2+^ concentrations ([Ca^2+^]_i_) were measured with a FlexStation 3 scanning fluorometer (Molecular Devices, Sunnyvale, CA, USA). Briefly, human neutrophils were suspended in HBSS^−^, loaded with Fluo-4AM at a final concentration of 1.25 μg/mL, and incubated for 30 min in the dark at 37 °C. After dye loading, the cells were washed with HBSS^−^, resuspended in HBSS^+^, separated into aliquots, and loaded into the wells of flat-bottom, half-area well black microtiter plates (2 × 10^5^ cells/well). To assess the direct effects of test compounds on Ca^2+^ flux, the compounds were added to the wells (final concentration of DMSO was 1%), and changes in fluorescence were monitored (λ_ex_ = 485 nm, λ_em_ = 538 nm) every 5 s for 240 s at room temperature after addition of the test compound. To evaluate inhibitory effects of the compounds on FPR1/FPR2-dependent Ca^2+^ flux, the compounds were added to the wells (final concentration of DMSO was 1%) with cells (human neutrophils or FPR1/FPR2 HL60 cells). The samples were preincubated for 10 min, followed by addition of 5 nM *f*MLF (for human neutrophils or FPR1-HL60 cells) or 5 nM WKYMVM (for FPR2-HL60 cells). The maximum change in fluorescence, expressed in arbitrary units over baseline, was used to determine the agonist response. Responses were normalized to the response induced by 5 nM *f*MLF or 5 nM WKYMVM, which were assigned as 100%. Curve fitting (at least five or six points) and calculation of median effective concentration values (EC_50_ or IC_50_) were performed by nonlinear regression analysis of the dose–response curves generated using Prism 7 (GraphPad Software, Inc., San Diego, CA, USA). Efficacy was determined by comparing individual responses activated by the test compounds to that induced by a positive control (5 nM *f*MLF), which was assigned a value of 100%.

#### 3.3.5. Chemotaxis Assay

Human neutrophils were suspended in HBSS^+^ containing 2% (*v/v*) heat-inactivated fetal bovine serum (2 × 10^6^ cells/mL), and effects of the compounds on *f*MLF-induced chemotaxis was analyzed in 96-well ChemoTx chemotaxis chambers (Neuroprobe, Gaithersburg, MD, USA). In brief, neutrophils were preincubated with the indicated concentrations of the test compounds or DMSO for 20 min at room temperature and added to the upper wells of the ChemoTx chemotaxis chambers. The lower wells were loaded with 30 µL of HBSS^+^ containing 2% (*v/v*) fetal bovine serum and the indicated concentrations of test sample plus 1 nM *f*MLF, DMSO (negative control), or 1 nM *f*MLF as a positive control. Neutrophils were added to the upper wells and allowed to migrate through the 5.0-µm pore polycarbonate membrane filter for 60 min at 37 °C and 5% CO_2_. The number of migrated cells was determined by measuring ATP in lysates of transmigrated cells using a luminescence-based assay (CellTiter-Glo; Promega, Madison, WI, USA), and luminescence measurements were converted to absolute cell numbers by comparison of the values with standard curves obtained with known numbers of neutrophils. Curve fitting (at least eight to nine points) and calculation of median effective concentration values (IC_50_) were performed by nonlinear regression analysis of the dose-response curves generated using Prism 5.

#### 3.3.6. Competition Binding Assay

Competition binding assays were performed to measure compound competition with the high-affinity fluorescent ligand WKYMVm-FITC for binding to human FPR1-HL60 cells, as described previously [38]. Briefly, FPR1-HL60 cells were preincubated with different concentrations of test compound for 30 min at 4 °C, followed by addition of 0.5 nM WKYMVm-FITC. After incubation for an additional 30 min at 4 °C, the samples were immediately analyzed using flow cytometry (LSRII, BD Biosciences, San Jose, CA, USA) without washing. The assay response range was defined by replicate control samples containing 1 µM of unlabeled *f*MLF (positive control) or buffer (negative control). The ligand competition curves were fitted by Prism software using nonlinear least-squares regression in a sigmoidal dose–response model to determine the concentration of added test compound that inhibited fluorescent ligand binding by 50% (i.e., IC_50_).

#### 3.3.7. ROS Production

ROS production was determined by monitoring L-012-enhanced chemiluminescence, which represents a sensitive and reliable method for detecting superoxide anion (O_2_^−.^) production. Human neutrophils were resuspended at 2 × 10^5^ cells/mL in HBSS^+^ supplemented with 40 µM L-012. Cells (100 µL) were aliquoted into wells of 96-well flat-bottomed microtiter plates containing test compounds at different concentrations (final DMSO concentration of 1%). Cells were preincubated for 10 min, and 200 nM PMA was added to each well to stimulate ROS production. Luminescence was monitored for 120 min (2-min intervals) at 37 °C using a Fluroscan Ascent FL microtiter plate reader (Thermo Electron, Waltham, MA, USA). The curve of light intensity (in relative luminescence units) was plotted against time, and the area under the curve was calculated as total luminescence. The compound concentration that inhibited ROS production by 50% of the PMA-induced response (positive control) was determined by graphing the percentage inhibition of ROS production versus the logarithm of concentration of test sample (IC_50_). Each curve was determined using five to seven concentrations.

#### 3.3.8. Assessment of Compound Cytotoxicity

Human promyelocytic leukemia HL60 cells were cultured in RPMI-1640 medium supplemented with 10% heat-inactivated FBS, 10 mM HEPES, 100 µg/mL streptomycin, and 100 U/mL penicillin. Cytotoxicity was analyzed with a CellTiter-Glo Luminescent Cell Viability Assay Kit (Promega, Madison, WI, USA), according to the manufacturer’s protocol. Briefly, wild-type HL60 cells were cultured at a density of 1 × 10^5^ cells/well with different concentrations of the compounds under investigation for 90 min at 37 °C and 5% CO_2_. Following treatment, the cells were allowed to equilibrate to room temperature for 30 min, substrate was added, and the samples were analyzed with a Fluoroscan Ascent FL microplate reader.

### 3.4. Molecular Modeling

Crown and aza-crown ethers are very conformationally flexible compounds. Therefore, a preliminary conformational search was performed for molecules **12**–**15**, and **19**, by the molecular mechanics method (MM+ force field) with the use of HyperChem 7 software. In this conformational search, all torsion angles inside the macrocycle and two exocyclic torsion angles about C-N bonds were varied. For compound **15**, the torsion angles about the carbon-carbon bonds of the O=C-CH_2_ fragments were additionally varied. Local geometry optimizations (5000) were made (10,000 optimizations for compound **15**), and 1000 conformations were kept within a 10 kcal/mol energy gap from the lowest energy conformation. Among these, 30 of the most optimal conformations (50 for molecule **15**) were selected, for which additional geometry optimizations were performed by the semiempirical PM3 method with HyperChem 7 program. Finally, 10 conformations of each aza-crown ether with the lowest PM3 energies were further optimized by the DFT method with the BLYP functional using the Gaussian 16 software (Gaussian, Inc., Wallingford CT).

In DFT calculations of macrocyclic ligands, complexes of Na^+^, K^+^, and Ca^2+^ with these ligands, and of the unbound ions, the def2-SVP basis set was used for C and H atoms. For N, O, Na^+^, K^+^, and Ca^2+^ participating in the formation of coordination bonds, the extended def2-TZVP basis set [58] was applied. Dispersion interactions were taken into account using the D3BJ model [59]. The attainment of the minimum on a potential energy surface for each investigated structure was confirmed by the absence of imaginary frequencies of normal vibrations. The binding energy in the gas phase on the formation of complexes was calculated by the equation:

ΔU = E(M^n+^·ligand) − [E(M^n+^) + E(ligand)], (3)

where E(M^n+^·ligand), E(M^n+^) (n = 1 or 2) and E(ligand) represent energies of the complex, metal ion, and free ligand, respectively, including thermal corrections to the energy at 298.15 K and corrections for zero-point vibrations [44]. In Equation (3), the values of E(M^n+^·ligand) and E(ligand) correspond to the complex and ligand in their lowest-energy conformations.

The basis set superposition error (BSSE), which is significant in the DFT calculations of coordination compounds, was taken into account using a counterpoise correction [60], as it was previously used for complexes of s-elements and transition metal ions with crown ethers (see, for example [43,44]). The DFT results were visualized using GaussView 6.

Compound physicochemical properties were computed using the SwissADME web server (http://www.swissadme.ch, accessed on 26 January 2021). Regression analyses were performed using STATISTICA 6 software.

## 4. Conclusions

We report the synthesis and analysis of twenty-four aza- and diaza-crown ethers with adamantyl, adamantylalkyl, 4-(aminomethyl)benzoyl, and ε-aminocaproyl substituents. This is the first report of the synthesis of compounds **10**, **11**, **14**, **15**, **19**, **22**, **23**, and **25**–**27**. Ten of the compounds (**8**, **10**–**17**, and **21**) caused a concentration-dependent increase in [Ca^2+^]_i_ in human neutrophils. On the other hand, compounds **8**, **10**, **13**–**17**, and **21** inhibited neutrophil activation by the chemotactic peptide *f*MLF, resulting in inhibition of *f*MLF-induced [Ca^2+^]_i_ flux. Some of these compounds also inhibited neutrophil ROS production and chemotaxis, as well as [Ca^2+^]_i_ flux in FPR-transfected HL60 cells. DFT analysis showed that Ca^2+^ ions bound more effectively to these crown ethers versus Na^+^ and K^+^. Moreover, DFT-optimized structures of the ligand-Ca^2+^ complexes and QSAR models demonstrated that the carbonyl oxygen atoms of these aza- and diaza-crown ethers participated in cation binding and can play an important role in Ca^2+^ transfer. Thus, this modeling provides a molecular basis to explain at least part of the ionophore mechanism of biological action of these crown ethers and may help elucidate mechanisms of Ca^2+^ transfer across neutrophil membranes facilitated by mobile ion carriers. Future studies are now in progress to evaluate the anti-inflammatory and immunomodulatory potential of these crown ethers.

## Figures and Tables

**Table 1:** Effect of aza- and diaza-crown ethers on [Ca^2+^]_i_ flux in human neutrophils.

Compound	EC_50_ (µM) (Efficacy, %)
**5**	N.A.
**6**	N.A.
**7**	N.A.
**8**	22.4 ± 4.3 (50)
**9**	N.A.
**10**	9.7 ± 2.6 (35)
**11**	15.6 ± 4.4 (40)
**12**	23.8 ± 7.6 (25)
**13**	6.9 ± 2.1 (25)
**14**	9.5 ± 2.7 (40)
**15**	4.7 ± 1.5 (110)
**16**	15.7 ± 3.8 (25)
**17**	15.9 ± 2.5 (50)
**18**	N.A.
**19**	N.A.
**20**	N.A.
**21**	11.3 ± 3.1 (40)
**22**	N.A.
**23**	N.A.
**24**	N.A.
**25**	N.A.
**26**	N.A.
**27**	N.A.
**28**	N.A.

N.A., no activity was found at concentration < 50 µM. Efficacy (in parentheses) is expressed as % of the response induced by 5 nM *f*MLF.

**Table 2:** Effect of aza- and diaza-crown ethers on N-formyl peptide receptor 1 or 2 (FPR1/FPR2) agonist-induced Ca^2+^ influx in human neutrophils and FPR-transfected HL60 cells and phorbol 12-myristate 13-acetate (PMA)-induced neutrophil reactive oxygen species (ROS) production.

Compound	FPR1/FPR2 Agonist-Induced Δ[Ca^2+^]_i_			PMA-Induced ROS Production IC_50_ (μM)
	Neutrophils IC_50_ (μM)	FPR1-HL60 IC_50_ (μM)	FPR2-HL60 IC_50_ (μM)	
**5**	N.A.	N.A.	N.A.	N.A.
**6**	N.A.	N.A.	N.A.	N.A.
**7**	N.A.	N.A.	N.A.	N.A.
**8**	2.6 ± 0.4	37.1 ± 4.8	N.A.	N.A.
**9**	N.A.	N.A.	N.A.	N.A.
**10**	11.2 ± 2.7	12.2 ± 1.9	36.4 ± 5.6	N.A.
**11**	9.4 ± 0.6	N.A.	29.1 ± 3.2	N.A.
**12**	4.0 ± 1.4	N.A.	N.A.	N.A.
**13**	7.4 ± 1.3	16.5 ± 3.2	32.6 ± 4.5	25.9 ± 2.7
**14**	3.5 ± 0.4	11.6 ± 2.1	8.3 ± 2.3	21.9 ± 2.2
**15**	4.1 ± 0.6	4.8 ± 0.9	7.2 ± 1.7	4.1 ± 0.1
**16**	3.4 ± 0.8	7.3 ± 2.6	30.7 ± 6.4	N.A.
**17**	1.5 ± 0.4	3.7 ± 0.7	7.6 ± 1.9	8.2 ± 0.1
**18**	N.A.	N.A.	N.A.	N.A.
**19**	N.A.	N.A.	N.A.	N.A.
**20**	N.A.	N.A.	N.A.	N.A.
**21**	10.2 ± 2.6	9.6 ± 2.1	28.2 ± 2.6	15.3 ± 1.8
**22**	N.A.	N.A.	N.A.	N.A.
**23**	N.A.	N.A.	N.A.	N.A.
**24**	N.A.	N.A.	N.A.	N.A.
**25**	N.A.	N.A.	N.A.	N.A.
**26**	N.A.	N.A.	N.A.	N.A.
**27**	N.A.	N.A.	N.A.	N.A.
**28**	N.A.	N.A.	N.A.	N.A.

N.A., no activity was found at concentration < 50 µM.

**Table 3:** Inhibitory effect of selected crown ethers on neutrophil chemotactic activity.

Compound	Chemotaxis, IC_50_, µM
**8**	13.2 ± 3.8
**11**	12.8 ± 5.3
**12**	15.7 ± 4.2
**13**	5.8 ± 1.4
**14**	6.1 ± 2.1
**15**	5.7 ± 0.2
**16**	5.3 ± 0.4
**17**	5.2 ± 1.7

**Table 4:** Characteristics of Na^+^, K^+^, and Ca^2+^ complexes with diaza-crown ethers calculated by DFT method.

Ligand	Cation (M^n+^)	NBO Charge at the Cation	Metal-Oxygen Distances, Å	Metal-Nitrogen Distances, Å	ΔU, kcal/mol
**12**	Na^+^	0.963	2.192…4.434	3.582	−71.06
	K^+^	0.979	2.576…4.916	4.008	−50.65
	Ca^2+^	1.814	2.302…2.444	2.673	−216.65
**13**	Na^+^	0.899	2.332…2.622	2.930, 3.030	−75.26
	K^+^	0.919	2.733…2.938	3.189, 3.130	−55.84
	Ca^2+^	1.813	2.295…2.490	3.092, 2.618	−239.26
**14**	Na^+^	0.886	2.316…2.621	3.146, 3.267	−81.89
	K^+^	0.924	2.733…3.007	3.267, 3.690	−59.46
	Ca^2+^	1.794	2.327…2.444	2.993, 3.055	−257.27
**15**	Na^+^	0.951	4.145 *, 2.257…2.507	3.581, 3.624	−79.30
	K^+^	0.967	4.499 *, 2.636…2.980	3.933, 3.997	−60.10
	Ca^2+^	1.818	2.674 *, 2.279…2.385	3.090, 2.837	−234.04
**19**	Na^+^	0.900	2.334…2.373	2.514, 2.516	−81.01
	K^+^	0.944	2.709…2.749	2.882, 2.885	−59.44
	Ca^2+^	1.790	2.316…2.382	2.482, 2.480	−240.81

* Distance from the central ion to the oxygen atom in the (CH_2_)_2_O(CH_2_)_2_ segment connecting nitrogen atoms of the macrocycle.

**Table 5:** The biological activities of aza-crown ethers and variables used in the regression analysis.

Compd.	MW	N_ha_	N_ar_	Csp^3^	N_rot_	N_HBA_	N_HBD_	MR	tPSA	iLog P	N_C=O_	N_Ad_	1/[EC_50_]
**5**	337.45	24	0	0.95	2	4	0	95	48.00	3.29	1	1	0
**6**	381.51	25	0	0.95	2	5	0	105.7	57.23	3.65	1	1	0
**7**	425.56	30	0	0.96	2	6	0	116.39	66.46	3.94	1	1	0
**8**	395.53	28	0	0.95	3	5	0	110.5	57.23	3.81	1	1	0.04464
**9**	439.59	31	0	0.96	3	6	0	121.2	66.46	3.83	1	1	0
**10**	429.55	31	6	0.72	2	5	0	121.61	57.23	3.69	1	1	0.10309
**11**	443.58	32	6	0.73	3	5	0	126.42	57.23	4.02	1	1	0.06410
**12**	498.7	36	0	0.93	4	4	0	147.2	59.08	4.36	2	2	0.04201
**13**	542.75	39	0	0.94	4	5	0	157.9	68.31	4.59	2	2	0.14493
**14**	586.8	42	0	0.94	4	6	0	168.6	77.54	4.73	2	2	0.10526
**15**	570.8	41	0	0.94	6	5	0	167.51	68.31	5.28	2	2	0.21277
**16**	586.8	42	0	0.94	4	6	0	168.6	77.54	5.31	2	2	0.06369
**17**	614.86	44	0	0.94	6	6	0	178.21	77.54	4.94	2	2	0.06289
**18**	367.52	26	0	1	2	5	0	105.5	40.16	3.72	0	1	0
**19**	514.78	37	0	1	4	5	0	157.5	34.17	4.72	0	2	0
**20**	558.84	40	0	1	4	6	0	168.2	43.40	5.74	0	2	0
**21**	586.89	42	0	1	6	6	0	177.81	43.40	6.44	0	2	0.08850
**22**	444.61	31	0	0.91	12	7	2	128.45	120.35	3.81	2	0	0
**23**	400.56	28	0	0.90	12	6	2	117.75	111.12	3.19	2	0	0
**24**	528.64	38	12	0.50	6	8	2	150.42	129.58	2.99	2	0	0
**25**	376.49	26	0	0.94	6	7	1	101.67	92.48	3.59	1	0	0
**26**	332.44	23	0	0.94	6	6	1	90.97	83.25	2.95	1	0	0
**27**	352.43	25	6	0.61	3	6	1	96.61	83.25	2.31	1	0	0
**28**	378.42	26	0	0.88	4	10	2	98.42	118.00	2.07	2	0	0

**Abbreviations**: MW, molecular weight (g/mol); N_ha_, number of heavy atoms; N_ar_, number of aromatic heavy atoms; Csp^3^, fraction of sp^3^-carbons among all carbon atoms; N_rot_, number of rotatable bonds; N_HBA_, number of H-bond acceptors; MR, molar refractivity (cm^3^/mol); tPSA, topological polar surface area (Å^2^); iLog P, lipophilicity (consensus Log Po/w); N_C=O_, number of carbonyl groups; N_Ad_, number of adamantyl groups.

## Data Availability

The data that support the findings of this study are available from the authors upon reasonable request.

## Sample Availability

Samples of the compounds are available from the authors.
